# Prevalence of Maternal Fever and Associated Factors among Postnatal Women at Kawempe National Referral Hospital, Uganda: A Preliminary Study

**DOI:** 10.3390/ijerph21030316

**Published:** 2024-03-08

**Authors:** Hilda Ainebyona, Elizabeth Ayebare, Allen Nabisere, Melissa A. Saftner

**Affiliations:** 1 Department of Nursing, Makerere University, Kampala P.O. Box 7072, Uganda; hildanbyona@gmail.com (H.A.); lizayeby@gmail.com (E.A.); nallenah25@gmail.com (A.N.); 2 School of Nursing, University of Minnesota, Minneapolis, MN 55455, USA

**Keywords:** postnatal fever, puerperal sepsis, factors associated with postnatal fever, Uganda

## Abstract

Fever is one of the most important signs of infection and can provide useful information for further assessment, diagnosis, and management. Early detection of postnatal fever could reduce severe outcomes, such as maternal mortality due to puerperal sepsis. The purpose of this cross-sectional study was to determine the prevalence of and associated factors of postnatal fever among postnatal women at Kawempe National Referral Hospital. Three hundred postnatal women were recruited. Temperature measurements were conducted and a 29-item questionnaire was completed along with the extraction of health history from the medical records of the participants. The prevalence of maternal fever was 58/300 (19.3%). Multivariable analysis indicated that only four factors—HIV-positive status (AOR = 2.56; 95% CI = 1.02–6.37), labor complications (AOR = 6.53; 95% CI = 2.40–17.71), prolonged labor (AOR = 3.12; 95% CI = 1.11–8.87), and more than 24 h spent in postnatal care (AOR = 5.16; 95% CI = 2.19–12.16)—were found to be significantly associated with postnatal fever. The prevalence of postnatal maternal fever among postnatal women at Kawempe National Referral Hospital was higher than that in other reports in the literature. The factors significantly associated with maternal fever were HIV-positive status, complications during labor, prolonged labor, and more than 24 h spent in postnatal care. Health workers involved in the provision of labor and obstetric services must follow guidelines to assess fever and manage the underlying conditions causing it.

## 1. Introduction

Infection or sepsis is the second leading cause of maternal mortality globally [1]. Maternal mortality is defined as the “number of female deaths from any cause related to or aggravated by pregnancy or its management (excluding accidental or incidental causes) during pregnancy and childbirth or within 42 days of termination of pregnancy, irrespective of the duration and site of the pregnancy [2]”. Maternal infections that can lead to sepsis include endometritis, urinary tract infection, wound infection, and phlebitis [3]. Maternal sepsis is a life-threatening condition defined as organ dysfunction caused by an infection during pregnancy, delivery, postnatal, or post-abortion period [4]. Sepsis resulting from childbirth and the postnatal period is largely preventable and can be reduced with good hygiene practices, early recognition of infection, and timely initiation of antibiotic treatment [1].

Mothers from low-income settings have the highest risk of sepsis and are more likely to develop poor outcomes than those in high-income settings are [5]. In Uganda, the maternal mortality ratio is estimated to be 189 per 100,000 live births [6,7]. Sepsis is the third leading cause of maternal deaths after hemorrhage and hypertensive disorders of pregnancy. Although in Uganda, the annual maternal and perinatal death surveillance and response report for 2022/2023 showed that sepsis contributed 9% of maternal deaths, facility-based studies have reported higher percentages [8]. Other significant causes of maternal mortality include hemorrhage, (40%), hypertensive disorders (16%), and indirect causes such as malaria or anemia (13%) [9]. One study attributed 24% of all maternal deaths to sepsis [6], while another study at a large tertiary hospital found it to be the largest contributor with 30.9% [10]. Alobo et al. reported that maternal mortality is highest among women living with HIV, those who over the age of 30, those who did not attend regular prenatal care appointments, those who were referred to higher level care late, and those who underwent delivery via cesarean section [6]. Although both studies found high rates of infection leading to maternal morbidity and mortality in Uganda, neither study described the prevalence of infectious signs such as fevers [6,10].

The burden of maternal mortality is substantial. Conceicao estimated that women who die lose approximately 39.8 to 41.5 years of life from childbirth-related causes [11]. Additionally, many women who narrowly survive a life-threatening pregnancy-related event often suffer from long-lasting complications. It has been estimated that for every single maternal death, approximately six women endure severe health issues, some of which may persist for a lifetime [12,13]. Given the high toll of maternal morbidity and mortality, target 3.1 of the Sustainable Development Goals (SDGs) sets the ambitious goal of reducing the global maternal mortality ratio (MMR) to below 70 maternal deaths per 100,000 live births by 2030 [14].

Postnatal maternal fever is one of the earliest signs of puerperal infections [15] and can indicate a need for further assessment, diagnosis, and management. Postnatal maternal fever is defined as a body temperature of 38.0 °C or higher occurring on any two of the first ten days after birth [16]. Fever is usually the first indicator of infection among postnatal women and can present within 24 h of birth. Postnatal maternal fever is associated with demographic and socioeconomic factors (e.g., maternal age, educational status, residential living area), obstetric factors (e.g., parity, timing and adequacy of antenatal visits, premature rupture of membranes (PROM), length of labor, mode and site of delivery), and medical co-morbidities (e.g., HIV, anemia, malaria, urinary tract infection). Early detection of postpartum maternal fever could reduce severe outcomes such as maternal mortality due to puerperal sepsis [17].

In a criterion-based audit study at one Ugandan referral facility, maternal temperature was checked for only 5.6% of women at admission to the postnatal ward at the baseline assessment [18]. The literature about postnatal maternal fever is limited, as most studies focus on postpartum infections in general. Unfortunately, even at the study hospital, a review of records for the last three years (2020–2022) revealed that a number of women returned to the hospital with puerperal sepsis soon after discharge, a condition that may have been prevented if their body temperature measurements had been taken during admission and prior to discharge [7].

This study was aimed at establishing the prevalence and factors associated with postnatal maternal fever, which will, in turn, help reduce the cases of puerperal sepsis. This is essential for designing measures for the prompt detection and management of maternal fever during and following childbirth, thereby contributing to a reduction in maternal morbidity and mortality.

## 2. Materials and Methods

### 2.1. Study Design

A quantitative, descriptive, cross-sectional study was conducted to determine the prevalence of postnatal maternal fever and related factors among postnatal women at Kawempe National Referral Hospital.

### 2.2. Setting and Sample

The study was conducted from the 13th to the 30th of October 2022 in the postnatal ward of Kawempe National Referral Hospital (NRH) in the capital city, Kampala. Kawempe NRH is the busiest public maternity unit in the country, with 20,485 births in the financial year of 2022/2023, which translates to an average of 57 births daily [18,19]. From July 2022 through June 2023, there were 1103 stillbirths reported at Kawempe Hospital and 125 maternal deaths. During the month of October, there were 1736 total births, which included 101 stillbirths and 12 maternal deaths. Nationally, Uganda reported 1.4 million deliveries in the country in the same time period and 1276 maternal deaths [9]. Kawempe NRH serves as a teaching hospital for medical and nursing schools and a national referral hospital for the surrounding catchment area. The maternity unit has 47 midwives, 20 specialist obstetricians and gynecologists, and a large number of residents that fluctuates depending on the enrollment at the universities that use the hospital for clinical placements.

Ugandan guidelines recommend a minimum of eight antenatal visits during pregnancy. Antenatal care occurs in the same building as labor and birth but in a separate, designated area. When in labor, women are first assessed and admitted to the labor ward. After birth, postnatal care is provided in the labor ward for the first two hours, and then the woman is transferred to the postnatal ward, where they stay for 24 h. According to the essential maternal and newborn clinical care guidelines for Uganda, every woman should be monitored for 24 to 48 h after a normal vaginal birth and at least 72 h after a cesarean section [16]. The guidelines recommend that postnatal women have vital signs, including temperature, monitored every 30 min in the first two hours and then every 6 h until 24 h after birth, as well as assessment of vaginal bleeding every hour for the first 6 h [16]. As in other Ugandan health facilities, it is not possible for all women to stay for 24 h due to the few admission beds and large number of women [20].

The participants included women who gave birth vaginally or via cesarean section and were admitted to the postnatal ward at Kawempe NRH. Those who were too ill or experienced a stillbirth were excluded. Those too ill to participate included women who were unable to talk or too weak to sustain an interview and those admitted to the high-dependency unit in the postnatal ward. Our focus was on routine postpartum women recovering from a normal vaginal delivery or cesarean section. A convenience sample of participants was consecutively recruited during the study period. Participants were approached at the postnatal ward after admission and invited to participate in the study. Those with interest received further information from the study staff, and written informed consent was obtained.

The sample size necessary to determine the prevalence of maternal fever for the study was determined with the Cochran formula using a 95% confidence level, a 5% margin of error, and an estimated prevalence of 30% [21,22]. The final sample size was determined to be 322 participants. In order to determine factors associated with maternal fever, the OpenEpi online calculator was used. In the Fleiss formula for unmatched case–control studies, we considered a two-sided confidence level of 95%, a power of 80%, and a ratio of cases to controls of 1:2. The hypothetical proportion of controls with exposure was estimated to be 51%, and the hypothetical proportion of cases with exposure was estimated to be 4.95%. The sample size was estimated to be 41 participants. Since the sample size for the prevalence of fever was higher than that of the associated factors, the sample size for prevalence was used to cater to both objectives. However, during the time of data collection, only 300 women admitted to the postnatal ward met the study’s inclusion criteria. This represented 93.2% of the desired sample size.

### 2.3. Study Variables

The dependent variables for this study included the prevalence of postnatal maternal fever, which was defined as the percentage of postnatal mothers with a body temperature of greater than or equal to 38.0 degrees Celsius in the first 10 days after delivery. The independent variables that were examined included socio-demographic factors, such as age, residential area, marital status, and educational background; obstetric factors, including parity, number of antenatal visits, length of labor, premature rupture of membranes, and the site of delivery, and medical conditions, such as urinary tract infections, HIV infection, anemia, malaria, and sexually transmitted infections (STIs).

The number of antenatal visits was the total number of visits related to pregnancy care for the mother. The length of labor was measured as the time taken from the onset of uterine contractions to the delivery of the placenta in hours, and the site of delivery was either a health center, which included any private or public clinics, maternity centers, or hospitals, or a home, which included any place other than a health center. Medical conditions, which included UTIs, HIV status, anemia, malaria, and STIs, were obtained from available medical records from the start of labor up to the recruitment date.

### 2.4. Data Collection Tools

An iHealth no-Touch Digital thermometer (Dongguan Yimai Industrial Chang’an Town, Dongguan City, China) was used to measure body temperature. The reliability of the thermometer was ensured by strictly following the user’s manual from the manufacturer [23]. The researchers collected data from the participants via a questionnaire administered by an interviewer that included the socio-demographic factors, obstetric factors, and medical factors listed in the previous section. The questionnaire was created based on the literature related to postnatal maternal fever, and experts in postnatal health reviewed the questionnaire for face validity. Additionally, the questionnaire was pre-tested with 10 postnatal women in Kawempe NRH.

### 2.5. Data Collection Procedure

The Makerere University School of Health Sciences Research and Ethics Committee approved this study (IRB: MAKSHSREC 2021-162). Additionally, the study was approved by the administration of Kawempe National Referral Hospital. Eligible postnatal women identified by staff and research team members were assured of confidentiality prior to their participation, and written informed consent was received prior to the study’s activities commencing. Participants were requested to have their temperatures taken, complete a 29-item questionnaire that included items about their demographics, pregnancy, and birth, and allow researchers to extract information from their medical records. The information extracted from the medical records included medical history, labor progress, and delivery information. The principal investigator read each question on the survey to the participants and entered their answers to ensure that literacy did not interfere with accurate data collection.

### 2.6. Data Analysis

The data collected were entered into the SPSS version 20 software and analyzed using STATA 15. There were no missing data because of our data collection methods, so all data were included in the analysis. Maternal fever was measured as a dichotomous variable, and its prevalence was described as a percentage (i.e., the total number of participants with postnatal maternal fever divided by the total number of participants in the study and then multiplied by 100 %). All independent variables were initially analyzed with a Chi-square test, and those with *p* < 0.2 were considered for multivariate logistic regression analysis. The level of significance was set at *p* < 0.05.

## 3. Results

### 3.1. Socio-Demographic Characteristics of the Respondents

The study was conducted among 300 postnatal mothers at Kawempe NRH, Kampala. The relevant demographic characteristics considered for the study were age, education level, number of children, occupation, and residence. Table 1 describes the demographic characteristics of the study respondents. The mean age of the study participants was 27 years, with the majority being above 24 years of age. Slightly more than half of the mothers had primary-level education, and very few (16.3%) had a post-secondary level of education. Most of the women were multiparous. Over half the women worked outside the home, and over three-quarters of the respondents lived in an urban setting.

### 3.2. Prevalence of and Factors Associated with Postnatal Maternal Fever

Fifty-eight of the 300 postnatal respondents had a fever, giving a prevalence of 19.3%. The Chi-square analysis showed that occupation (*p* = 0.136) and residence (*p* = 0.015) were the only socio-demographic factors that were associated with maternal fever among postnatal women with the *p*-value being set to <0.2 (see Table 2). Fever was more common in women who were over 24 years of age, those who had not advanced past a primary level of education, and those who identified as homemakers or unemployed (53.5%).

As indicated in Table 3, the only medical factors significantly associated with maternal fever were HIV (*p* = 0.020), malaria (*p* = 0.020), palmar pallor (*p* = 0.023), and conjunctival pallor (*p* = 0.016). STI status (*p* = 0.504) was not significantly associated with maternal fever. Labor complications (*p* ≤ 0.001), length of labor (*p* ≤ 0.001), number of vaginal examinations (*p* = 0.005), infection signs during labor (*p* ≤ 0.001), mode of delivery (*p* = 0.015), and time spent after labor (*p* ≤ 0.001) were significantly associated with postnatal fever (see Table 4). The number of ANC visits (*p* = 0.306) and gestational age (*p* = 0.470) were not significantly associated with postnatal maternal fever.

It was noted in Table 4 that fever was more common in women who had experienced complications during labor than in those who had not (*p* < 0.001). Additionally, fever was more common in those who had greater than three vaginal examinations during labor (*p* = 0.005). Fever was also more common in women who were in the hospital for 24 h or more postnatally (*p* < 0.001).

### 3.3. Multivariate Logistic Regression Analysis of the Factors Associated with Maternal Postnatal Fever

Factors with a *p*-value of less than 0.2 from the bivariate analysis were subjected to multivariable logistic regression analysis to obtain the adjusted odds ratios (AORs) and the corresponding 95% CI. Table 5 shows that none of the socio-demographic factors were significantly associated with postnatal maternal fever. HIV-positive status (*p* = 0.045) was the only medical factor that was significantly associated with postnatal fever. The obstetric factors that were significantly associated with postnatal fever were labor complications (*p* < 0.001), prolonged labor (*p* = 0.031), and time spent in postnatal care (*p* < 0.001)

## 4. Discussion

Postnatal fever is a major indicator of postnatal illness and is a common symptom of puerperal sepsis [24,25,26]. Health workers must ensure that they take the body temperatures of postnatal mothers at admission to the postnatal ward and before discharging them to ensure that potential postnatal infections are ruled out or diagnosed early. This allows for the effective treatment and prevention of wasted time and financial resources while reducing maternal mortality.

The analysis indicated that those who were HIV-positive (AOR = 2.56; 95% CI = 1.02–6.37) were about three times more likely to have postnatal fever than those who were HIV-negative. The results also indicated that those who experienced complications during labor (AOR = 6.53; 95% CI = 2.40–17.71) were approximately seven times more likely to have postnatal fever than those who did not experience labor complications. Additionally, those with prolonged labor (AOR = 3.12; 95% CI = 1.11–8.87) were about three times more likely to experience postnatal fever than those who did not experience prolonged labor. Finally, women admitted to the postnatal ward for longer than 24 h at the time of temperature measurement (AOR = 5.16; 95% CI = 2.19–12.16) were five times more likely to be diagnosed with postnatal fever than those who had spent less than 24 h in the hospital.

Those who were HIV-positive were approximately three times more likely to have postnatal fever than those who were HIV-negative. This was consistent with a study completed in South Africa that found that women with HIV had over three times the risk of puerperal sepsis compared to those without HIV. This increased risk was attributed to the mothers’ compromised immune status and generally poor health, leaving women living with HIV more vulnerable to infections [27]. In addition, in a study conducted in Mbarara Regional Referral Hospital in Uganda, HIV was found to be associated with an increased risk of postnatal complications such as uterine rupture and PROM, which were found to be significantly associated with postnatal fever in this study [28].

This study’s results also indicated that those who experienced complications during labor were about seven times more likely to have postnatal fever than those who did not experience labor complications, and those who had experienced prolonged labor were about three times more likely to have postnatal fever than those who had not experienced prolonged labor. This was likely because labor complications such as prolonged labor, obstructed labor, and PROM are risk factors for ascending infections, which often present with postnatal fever [29]. Similarly, one study in Nigeria found an association among PROM, prolonged labor, and poor maternal health outcomes, including an increased risk of puerperal sepsis, which presented with postnatal fever in 90.5% of the women [30]. Another study in Ethiopia found that those who experienced prolonged labor were three times more likely to develop puerperal sepsis, which was similar to the rate of fevers in this study [31].

Additionally, it is important to consider variables that may contribute to postnatal fever, including the frequency and number of vaginal exams that a woman may experience during labor. Gluck et al. noted that the number of vaginal exams that a woman has during labor is significantly related to febrile morbidity [32]. Although not tracked in this study, it would be important for future studies to consider the number of vaginal exams that a woman experiences during the first and second stages of labor and fever outcomes. However, all clinicians should consider the necessity of routine vaginal exams, particularly during uncomplicated labor.

The duration of one’s stay in the postnatal ward was associated with postnatal fever. This was consistent with the work of Ngonzi et al., who found that the main cause of postnatal fever in Uganda was puerperal sepsis [10]. Signs of puerperal sepsis usually appear more than 24 h after birth [25]. Therefore, healthcare givers should ensure close monitoring of mothers’ body temperatures even several days after birth for the timely detection of fever and, thus, timely diagnosis of postnatal infections such as puerperal sepsis. This finding is in line with the findings of a study conducted in Cameroon, which stated that postnatal fever was more common in the first 3 postnatal days, excluding the first 24 h [33]. The same study also emphasized that the timing of the onset of fever is an important management strategy for healthcare providers for the timely management of postnatal infections and other complications. It is important to note that at Kawempe Hospital, those who stayed longer than 24 h often underwent delivery via cesarean section or had neonates that were admitted to the neonatal intensive care unit. Having a cesarean section increases the risk of postnatal infection. Therefore, it is even more critical to ensure effective screening for this group of women.

In this study, the prevalence of maternal fever among postnatal women at Kawempe NRH was 19.3%. Of every 100 mothers who delivered at the hospital, approximately one-fifth developed a postnatal fever. This rate of postnatal maternal fever is much higher than the 5–7% reported by Anbazhagan and Harper [34] and the 5% reported at Mbarara Regional Referral Hospital [10]. These differences in findings may be due to variations in the study setting and sample size.

The current study was carried out in a national referral hospital, which has the highest volume and acuity of patients in Uganda. This higher acuity may impact the overall risk of fever. Additionally, the higher rate of cesarean sections at this national referral hospital compared to that in lower-level health centers may also inflate the postnatal fever rates. Cesarean sections increase the rate of overall infections five-fold [35]. It is also possible that the current study found a much higher prevalence of fever among postnatal mothers because the sample size was smaller than that in previous studies, and point-prevalence was used, unlike in the study by Ngonzi, which was longitudinal [10]. Future studies will need to be conducted to confirm the findings of this study. More importantly, quality improvement work could occur immediately to ensure that all women are postnatally screened for fever in accordance with the national guidelines.

### Limitations

This study involved the provision of sensitive information by the study participants, which could have led to them concealing information. This was addressed by assuring the participants of confidentiality, collecting data in a private space, and limiting access to the collected data to only authorized personnel. A further limitation was that only 300 postnatal mothers met the criteria for inclusion in the study. Therefore, a smaller sample size of 300 was used, as opposed to the calculated 322. Nonetheless, we believe that this sample size of 300, which was 93.2% of the expected sample size, was sufficient for the realization of the objectives of the study. Finally, the data were collected during a short window of time in October 2022, which could have impacted their generalizability.

## 5. Conclusions

The prevalence of postnatal maternal fever among postnatal women at Kawempe National Referral Hospital was very high at 19.3%. The factors that were significantly associated with maternal fever were positive HIV status, complications during labor, prolonged labor, and more than 24 h spent in postnatal care. Therefore, midwives and other health workers involved in the provision of labor and obstetric services must prevent or manage these conditions in a timely manner in order to prevent poor maternal outcomes.

With the high prevalence of postnatal fever found in this study, health workers should take a woman’s body temperature prior to discharge as a standard of care. Health workers should also ensure that postnatal women whose body temperature is found to be high (38 °C and above) are further examined to identify and treat underlying conditions before discharge. Midwives and other healthcare providers should ensure the prevention and or timely management of any complications during labor to prevent postnatal complications. Infection prevention measures, including prophylactic antibiotics as indicated, consistent hand hygiene, and the use of sterile gloves, should be the standard of care for women giving birth. Further research should be completed to assess infections and determine their relationships with maternal fever.

## Figures and Tables

**Table 1:** Demographic characteristics of the respondents.

Characteristic	Frequency (N = 300)	Percent %
Age (years)		
24 years and below	82	27.3
Above 24 years	218	72.7
Formal Education Level		
Primary	167	55.7
Secondary	84	28.0
Post-Secondary	49	16.3
Number of Children		
1	70	23.3
2–4	181	60.3
5 or more	49	16.3
Occupation		
Employed	166	55.3
Housewife/unemployed	134	44.7
Residence		
Rural	34	11.3
Urban	266	88.7

**Table 2:** Socio-demographic factors associated with maternal fever among postnatal women at Kawempe National Referral Hospital.

Variable	No Fever (<38.0 °C) n (%)	Fever (≥38.0 °C) n (%)	Total N (%)	Crude Odds Ratio [95% CI]	*p*-Value
Age (complete years)					
≤24 years	68 (28.1)	14 (24.1)	82 (27.3)	0.81 [0.42, 1.58]	0.544
25 and above	174 (71.9)	44 (75.9)	218 (72.7)	1	
Formal education level					
Primary	131 (54.1)	36 (62.1)	167 (55.7)	3.85 [0.49, 30.25]	0.200
Secondary	97 (40.1)	21 (36.2)	118 (39.3)	1	
Post-secondary	14 (5.8)	1 (1.7)	15 (5.0)	1	
Number of Children					
1	59 (24.4)	11 (19.0)	70 (23.3)	0.70 [0.34, 1.47]	0.346
2–4	143 (59.1)	38 (65.5)	181 (60.3)	1	
More than 4	40 (16.5)	9 (15.5)	49 (16.3)	1	
Occupation					
Employed	139 (57.4)	27 (46.5)	166 (55.3)	1	
Housewife/unemployed	103 (42.6)	31 (53.5)	134 (44.7)	1.55 [0.73, 2.76]	0.136 *
Residence					
Rural	22 (9.1)	12 (20.7)	34 (11.3)	2.61 [1.21, 5.64]	0.015 *
Urban	220 (90.9)	46 (79.3)	266 (88.7)	1	

* indicates a *p*-value of <0.2.

**Table 3:** Medical factors associated with maternal fever among postnatal women at Kawempe National Referral Hospital.

Variable	No Fever (<38.0 °C) n (%)	Fever (≥38.0 °C) n (%)	Total N (%)	Crude Odds Ratio [95% CI]	*p*-Value
HIV status					
Negative	209 (86.72)	43 (74.14)	252 (84.28)	1	
Positive	32 (13.28)	15 (25.86)	47 (15.72)	2.28 [1.14, 4.57]	0.020 *
Malaria infection					
Yes	5 (2.08)	5 (8.77)	10 (3.37)	4.52 [1.26, 16.18]	0.020 *
No	235 (97.92)	52 (91.23)	287 (96.63)	1	
Sexually Transmitted Infection					
Yes	8 (3.32)	3 (5.17)	11 (3.68)	1.59 [0.41, 6.18]	0.504
No	233 (96.68)	55 (94.83)	288 (96.32)	1	
Conjunctiva Pallor					
Yes	14 (5.79)	9 (15.52)	23 (7.67)	2.99 [1.23, 7.30]	0.016 *
No	228 (94.21)	49 (84.48)	227 (92.33)	1	
Palmar Pallor					
Yes	15 (6.20)	9 (15.52)	24 (8.00)	2.78 [1.15, 6.72]	0.023 *
No	227 (93.60)	49 (54.48)	276 (92.00)	1	

* indicates a *p*-value of <0.05.

**Table 4:** Obstetric factors associated with postpartum maternal fever among women at Kawempe National Referral Hospital.

Variable	No Fever (<38.0 °C) n (%)	Fever (≥38.0 °C) n (%)	Total N (%)	Crude Odds Ratio [95% CI]	*p*-Value
Antenatal Care Visits					
0–3	103 (42.6)	29 (50.0)	132 (44.0)	1	
At least 4	139 (57.4)	29 (50.0)	168 (56.0)	1.35 [0.76, 2.40]	0.306
Labor complications					
Yes	46 (19.0)	40 (69.0)	86 (28.6)	9.47 [4.98, 18.00]	<0.001 **
No	196 (81.0)	18 (31.0)	214 (71.3)	1	
Length of Labor (hours)					
<18 h	218 (90.1)	29 (50.0)	247 (82.3)	1	
18 h or more	24 (9.9)	29 (50.0)	53 (17.7)	9.08 [4.67, 17.67]	<0.001 **
Number of vaginal examinations					
1–3	134 (55.4)	20 (34.5)	154 (51.3)	1	
>3	108 (44.6)	38 (65.5)	146 (48.7)	2.36 [1.30, 4.29]	0.005 **
Signs of infection during labor					
Yes	19 (7.9)	18 (31.0)	37 (12.3)	5.28 [2.55, 10.93]	<0.001 **
No	223 (92.1)	40 (69.0)	263 (87.7)	1	
Birth mode					
SVD	161 (66.5)	28 (49.1)	189 (63.2)	1	
C/S	81 (33.5)	29 (50.9)	110 (36.8)	2.06 [1.15, 3.69]	0.015 *
Gestational age					
<38	29 (12.0)	5 (8.6)	34 (11.3)	0.69 [0.26, 1.88]	0.470
≥38	213 (88.0)	53 (91.4)	266 (88.7)	1	
Time spent postpartum					
<24 h	194 (80.2)	23 (39.7)	217 (72.3)	1	
≥24 h	48 (19.8)	35 (60.3)	83 (27.7)	6.15 [3.33, 11.36]	<0.001 **

* indicates a *p*-value of <0.05; ** indicates a *p*-value of <0.01.

**Table 5:** Multivariate logistic regression analysis of the factors associated with maternal postpartum fever among postnatal women at Kawempe National Referral Hospital.

Factor	Crude Odds Ratio (95% CI)	*p*-Value	Adjusted Odds Ratio (95% CI)	*p*-Value
**Socio-demographic factors**				
No employment	1.55 (0.73, 2.76)	0.136	1.78 (0.81, 3.89)	0.151
Rural residence	2.61 (1.21, 5.64)	0.015	1.77 (0.63, 4.98)	0.277
**Medical factors**				
HIV-positive	2.28 (1.144.57)	0.020	2.56 (1.02, 6.37)	0.045 *
Malaria-positive	4.52 (1.26, 16.18)	0.020	3.16 (0.58, 17.22)	0.183
Palmar pallor present	0.36 (0.15, 0.87)	0.023	0.65 (0.82, 5.09)	0.679
Conjunctival pallor present	2.99 (1.23, 7.30)	0.016	2.90 (0.34, 24.85)	0.332
**Obstetric factors**				
Labor complications ^β^	9.47 (4.98, 18.00)	<0.001	6.53 (2.40, 17.71)	<0.001 **
Prolonged labor	9.08 (4.67, 17.67)	<0.001	3.12 (1.11, 8.87)	0.031 *
Vaginal exams	2.36 (1.30, 4.29)	0.005	0.53 (1.89, 1.47)	0.219
Mode of birth	2.06 (1.14, 3.69)	0.015	0.554 (0.23, 1.34)	0.189
Time after birth >24 h	6.15 (3.33, 11.36)	<0.001	5.16 (2.19, 12.16)	<0.001 **
Infection signs during labor	5.28 (2.55, 10.93)	0.005	2.44 (0.95, 6.30)	0.065

^β^ includes obstructed labor, prolonged labor, intrapartum hemorrhage, and uterine rupture. * indicates a *p*-value of <0.05; ** indicates a *p*-value of <0.01.

## Data Availability

The data are available upon request to the corresponding author.
